# The Significance of Biomechanics and Scaffold Structure for Bladder Tissue Engineering

**DOI:** 10.3390/ijms222312657

**Published:** 2021-11-23

**Authors:** Marta Hanczar, Mehran Moazen, Richard Day

**Affiliations:** 1 Applied Biomedical Engineering Group, Centre for Precision Healthcare, UCL Division of Medicine, University College London, London WC1E 6JF, UK; marta.hanczar.18@ucl.ac.uk; 2 UCL Department of Mechanical Engineering, University College London, London WC1E 7JE, UK; m.moazen@ucl.ac.uk

**Keywords:** reconstructive urology, regenerative medicine, bladder biomechanics, biomaterials, urinary bladder, scaffold structure

## Abstract

Current approaches for bladder reconstruction surgery are associated with many morbidities. Tissue engineering is considered an ideal approach to create constructs capable of restoring the function of the bladder wall. However, many constructs to date have failed to create a sufficient improvement in bladder capacity due to insufficient neobladder compliance. This review evaluates the biomechanical properties of the bladder wall and how the current reconstructive materials aim to meet this need. To date, limited data from mechanical testing and tissue anisotropy make it challenging to reach a consensus on the native properties of the bladder wall. Many of the materials whose mechanical properties have been quantified do not fall within the range of mechanical properties measured for native bladder wall tissue. Many promising new materials have yet to be mechanically quantified, which makes it difficult to ascertain their likely effectiveness. The impact of scaffold structures and the long-term effect of implanting these materials on their inherent mechanical properties are areas yet to be widely investigated that could provide important insight into the likely longevity of the neobladder construct. In conclusion, there are many opportunities for further investigation into novel materials for bladder reconstruction. Currently, the field would benefit from a consensus on the target values of key mechanical parameters for bladder wall scaffolds.

## 1. Introduction

### 1.1. The Need for a Biomaterials-Based Approach to Bladder Reconstruction

The urinary bladder is a complex muscular organ that undergoes cyclic mechanical changes to facilitate storing and voiding urine. Multiple conditions can affect normal bladder function, including cancer (400 million people worldwide [1]), neurogenic conditions, radiation injury, intractable incontinence and congenital anomalies [2,3].

The treatment of these disorders includes interventions by partial (augmentation cystoplasty) or whole bladder (neobladder) reconstruction in order to regain urine storage and voiding functions. While robot-assisted surgery can lower the risk of some complications [4], a successful outcome is often dependent on the material used for this reconstruction.

Commonly, gastrointestinal segments obtained from the patient during the surgery are used. While this approach offers many advantages, such as the easy formation of hollow structures, pre-vascularization of the tissue and autologous origin [5], there are also many disadvantages. These are caused mainly by the intestine metabolite reabsorption and mucous secretion [6] contributing to urinary tract infection, bladder perforation, carcinogenesis and many others [2,3,7].

These complications lead to major morbidity including a decrease in quality of life for both young and elderly patients [2]. Especially affected are geriatric patients for which metabolic disorders and the major invasiveness of the procedure can be life-endangering [8].

These factors create an urgent need for cost-effective and robust regenerative therapies to replace the usage of gastrointestinal segments with biomaterials [3,9]. Tissue engineering is an optimal approach to creating such materials since it enables adjusting the properties of biomaterials to correspond to the needs of the replaced organ [9].

### 1.2. Unmet Needs for Bladder Reconstruction

The first attempts to find alternatives to gastrointestinal segments for bladder replacement started in the 1950s and 1960s with failed clinical trials using plastic bladder urinary substitutes [10,11,12]. Since then, many more advanced materials have been developed and tested in clinical trials. These included materials, such as gelatine sponge [13,14], Nobecutane [15], Japanese rice paper [16], lyophilised human dura patch [17,18], bovine pericardium [19] and porcine small intestinal submucosa [20]. However, many of the materials investigated still resulted in unsatisfactory effects due to poor mechanical integrity, limited mucosal regrowth, extensive fibrosis [21] and limited vasculature growth [22]. Many of these trials failed phase II due to serious complications, such as bladder perforation, associated with decreased scaffold compliance [3,23]. These failures have been attributed to the underestimation of the bladder complexity [23].

Therefore, current research employs more profound analyses of the developed materials and performs multiple tests on large animal models, such as pigs or dogs, before proceeding into trials on humans. This research often focuses on aiming to replicate the histological appearance of the native bladder and the interaction of single-cell types with the scaffold [24].

Important histological structures of the bladder tissue are required for its key functions, mainly urine storage and voiding. While recreation of the histological structure can facilitate function, reaching the appropriate histology might be impaired due to the biomaterials not being able to facilitate barrier function during the initial stages of biomaterial implantation into the host. For example, urine leakage through the scaffold is known to result in toxicity [25,26]. This can potentially lead to an unfavourable immune reaction and fibrosis leading to biomaterial failure. This could be prevented by paying greater attention to the biomechanical properties and scaffold structure, as discussed later in the review.

Additionally, there are circumstances in urologic surgery where completely functional bioengineered bladder tissue is not expected or necessary. In particular, the aim of performing the augmentation cystoplasty in patients with spina bifida is to primarily increase the bladder capacity and decrease detrusor contractility in order to preserve upper urinary tract function [7,27]. Therefore, the requirements for the bioengineered constructs might not be that strict regarding the histological appearance and other cellular aspects. Therefore, the main goal for the studies that create biomaterials could be to facilitate the bladder function by ensuring the elasticity and impermeability of the constructs.

As described above, many properties, such as mechanical strength, elasticity and impermeability of the construct are key to achieving long-term stability and function of the bioengineered construct [21]. Various efforts have been made in the field to address these properties. This review aims to evaluate the mechanical properties of the bladder wall and how the current research on materials used in bladder bioengineering has addressed these biomechanical challenges.

## 2. Methods

The review hereby presented is not a systematic review. The PubMed database was searched for results of bladder tissue mechanical testing and results were compared with other papers on the subject. To ensure high quality of the data collected on the biomechanical properties of the materials used for bladder engineering the PubMed database was searched using the search query ((((((((regenerative medicine) OR (biomaterials)) OR (biomaterials science)) OR (biomaterials tissue engineering)) OR (tissue scaffolds [MeSH Terms])) OR (regenerative medicine [MeSH Terms])) OR (Biocompatible Materials [MeSH Terms])) AND (((bladder) OR (lower urinary tract)) OR (Urinary Bladder [MeSH Terms])) AND (((biomechanical properties) OR (biomechanics)) OR (Biomechanical Phenomena [MeSH Terms])). The papers were selected on the usage of uniaxial testing methods, as well as reporting of strain and stress measures with standard deviation measured in percentage and MPa respectively. Additionally, the PubMed database was searched for studies performing urodynamic testing on biomaterial types, which lacked mechanical quantification in uniaxial tests. The selected studies were compared to other reviews on the topic of materials in bladder engineering.

## 3. Bladder Structure

To understand and determine the desired qualities of such bioengineered constructs, it is essential to understand the histology and the biomechanics of the bladder. This section will focus on presenting the histological features of the bladder that facilitate its function and that are relevant to biomaterials.

**Figure 1:**
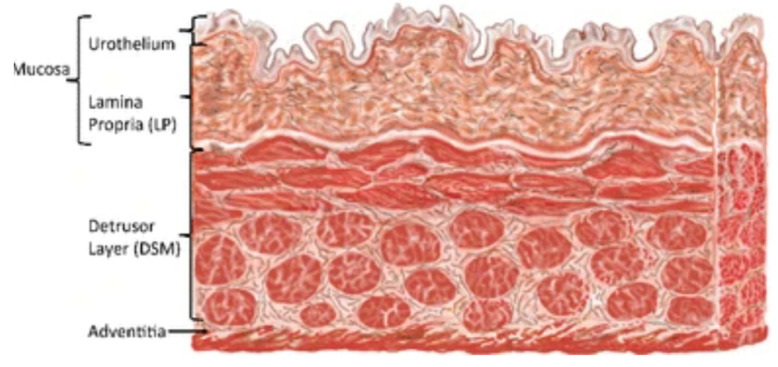
Diagram of the bladder wall structure. Figure modified and permission obtained from [30].

The bladder is a hollow muscular structure that can change its size and shape to accommodate up to 800 mL of urine [28]. The lumen of the bladder is highly wrinkled to maintain a barrier function while loading and voiding. The bladder wall is largely made up of four layers: urothelium, lamina propria, muscularis propria (detrusor) and adventitia [29], as seen in Figure 1.

The urothelium is the closest to the lumen of the bladder. This stratified epithelium consists of a single apical layer of umbrella cells, 2–3 layers of polygonal cells (intermediate) and 2–3 layers of cuboidal cells (basal). Umbrella cells are connected via tight junctions and covered in a glycoprotein layer–uroplakin, that acts as a protective barrier against an unfavourable bladder environment [25,31]. Its harmful components include urine, which was proven to have cytotoxic activity [3,26,32], reduced blood perfusion and microbial pathogens [3]. The lack of this barrier, simulated by uroplakin gene knock-out, results in increased water and urea permeability, detrusor muscle failure and more sex-specific bladder dysfunction [29,33,34].

**Figure 2:**
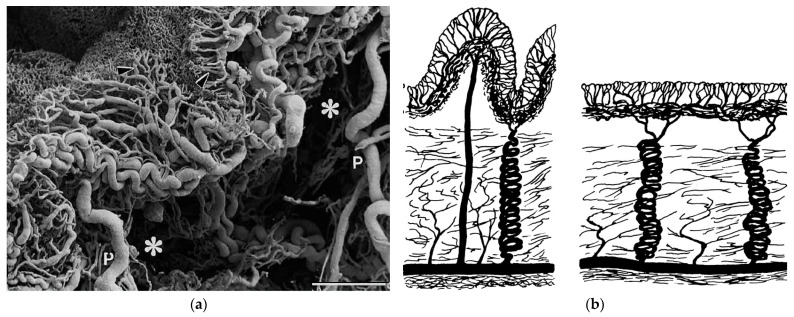
Diagram of the bladder vessel microarchitecture. (**a**) Shows the profile of the mucosal layer vasculature obtained via SEM. P—perpendicular vessels originating from the adventitial plexus, *—submucosa. (**b**) Graph showing structure of the vasculature in non distended (**left**) and distended bladder (**right**). Figures obtained with permission from [37].

Many features of the bladder prevent structural damage caused by bladder distension. This can be seen in the urothelium, which has the capability to reorganize to a smaller number of layers as the bladder wall stretches [31]. Another feature is highly elastic vasculature that prevents an increase in vascular pressure during bladder filling [35]. Additionally, the vasculature shows an explicit tortuous architecture that facilitates the bladder’s contraction without the risk of damage to the blood vessels [36,37]., as seen in Figure 2. Lastly, fibrillar collagens in the lamina propria layer enable regaining the previous structure and size after bladder distension [29,36].

As seen above, many major histological characteristics exist to eliminate the harm caused by constant distension and contraction and preserve the barrier function during this behaviour. Surprisingly, there is a significant lack of acknowledgement regarding the importance of this mechanism in many of the biomaterials that have been investigated to date, as described later in the review.

## 4. Biomechanical Properties of the Bladder

Regenerative medicine for bladder engineering aims to create materials that will foster tissue regeneration and support the mechanical function of the bladder until the completion of regeneration. Both processes rely on the biomechanical qualities of the materials. The physical stimuli of the biomaterial can influence the proper morphogenesis, maintenance and repair of tissues [38]. Additionally, the biomechanical properties of the material should enable constant cycles of contraction and distension while preventing urine leakage and deformation of the scaffold [21]. Therefore, it is crucial to investigate the properties of the bladder, understand their role and manipulate them to improve the regeneration and function of the tissue. Studies to investigate the inherent mechanical properties of the bladder started in the early 70s when it was confirmed that the bladder has viscoelastic properties [39], which can be further described using physical terms common in describing biomechanics of soft tissues. Several key engineering terms are used to describe the biomechanical properties of the bladder wall:

### 4.1. Strain

**Figure 3:**
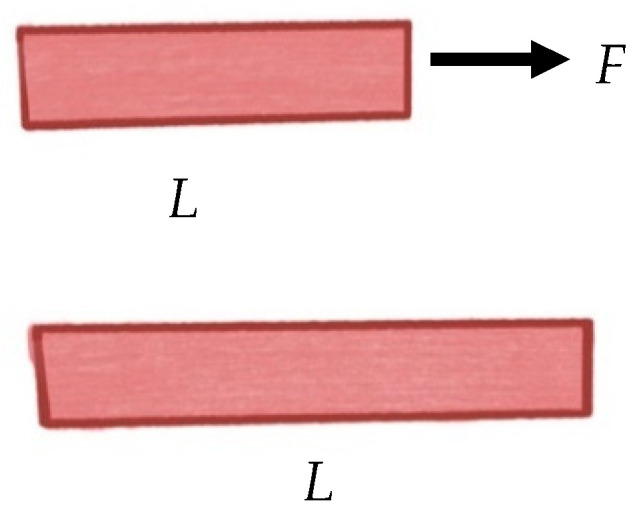
Strain illustrated.

Strain is the measure of deformation caused by the forces applied to a material. In 2D, this is defined as changes of the length over the initial length $\varepsilon =\frac{\left(L-L_{0}\right)}{L_{0}}$, where *L*_0_ is the initial length of the tissue sample and *L* is the length of the bladder strip after the force (*F*) is applied [40], as seen in Figure 3. Note, the strain does not have a unit, i.e., it is dimensionless.

### 4.2. Stress

**Figure 4:**
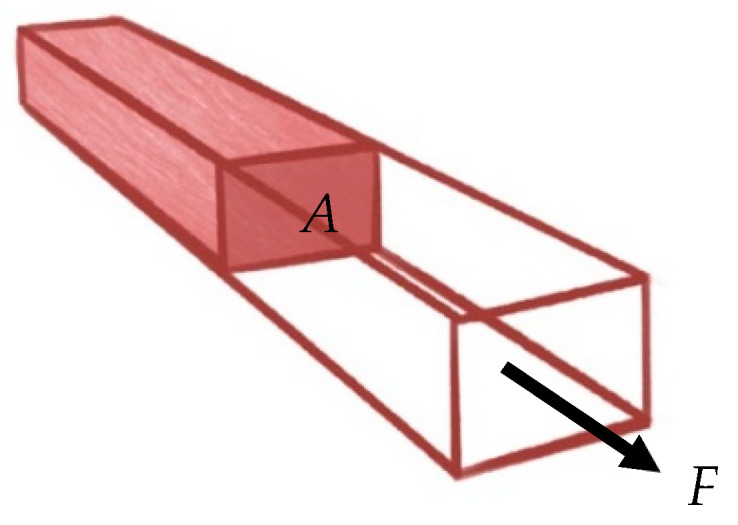
Stress illustrated.

Stress is defined as force per unit area of the material, $\sigma =\frac{F}{A}$, where *F* is the external force that acts on the tissue and *A* is the cross-sectional area of the tissue, as seen in Figure 4. This value is reported in Pascals (Pa, i.e., N/m^2^) [40].

### 4.3. Stress-Strain Curve

**Figure 5:**
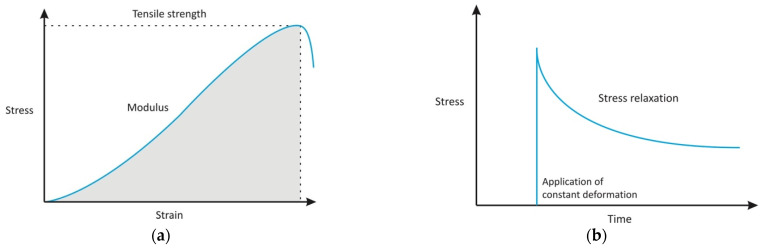

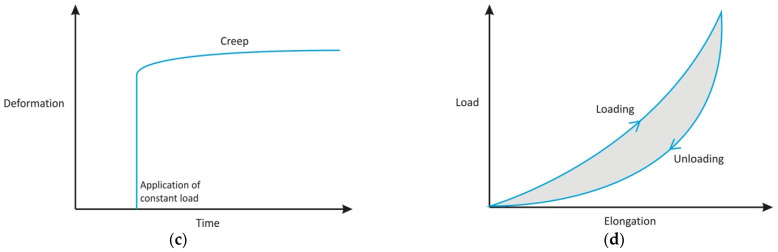
(**a**) Shows stress-strain curve. (**b**–**d**) Shows the schematic diagrams characterising tissues with viscoelasticity. (**b**) Shows relaxation. (**c**) Shows creep in this context deformation is meant as strain. (**d**) Shows the hysteresis loop. Elongation is meant as strain value. Figures obtained with permission from [41].

The stress-strain curve is the relationship between the aforementioned two qualities. It is obtained by plotting the measure of deformation for increasing the amount of force applied on the material, as seen in Figure 5a. The relation of those two qualities can reveal important information about the material, such as ultimate tensile strength–highest stress until material rupture.

### 4.4. Viscoelasticity

However, the bladder due to its compositional complexity, i.e., a mixture of fluid and solid materials, display stress-strain behaviours that also vary pending the timing and speed of loading as well as its magnitude. This characteristic of the materials (and bladder) is called viscoelasticity [21].

Viscoelastic materials display stress relaxation that is a phenomenon when a biological sample is suddenly strained and while the strain is maintained at a constant deformation, the corresponding stress in the sample will decrease with time, shown in Figure 5b. Similarly, if the sample is suddenly stressed and the applied stress is maintained at a constant level the sample continues to deform, which is described as creep, shown in Figure 5c. Additionally, the stress-strain relationship shows different strain values during loading of the sample, this is when the stress is increased, compared to during unloading, the stress exerted on it is lowering. This phenomenon is called hysteresis and is demonstrated in Figure 5d [40].

The experimental data collected during mechanical studies are usually represented in behavioural curves, as seen in Figure 5d. These are difficult to use for comparison because of the lack of simple quantification. Therefore, many mathematical models are developed to characterize and predict the viscoelastic behaviour of the tissues [42,43]. Comparison of collected data to previously created models enables researchers to extract comparable parameters of their results [40]. Unfortunately, due to the inadequacy of the models [21], this practice is not currently used in this field.

### 4.5. Preconditioning

**Figure 6:**
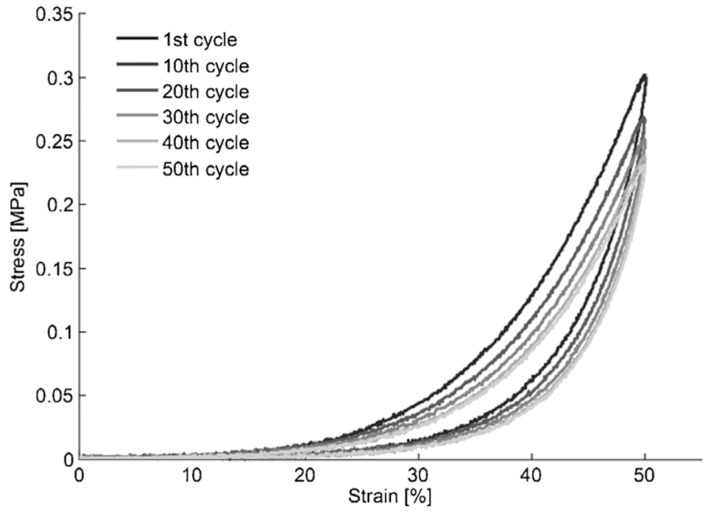
Represents the hysteresis and the load-history dependant behaviour in a one-year-old pig bladder. The cycle numbers are indicated by different shades of grey represented in the figure legend. The peak stress and the figure shapes stabilised after 50 cycles. Figure obtained with permission from [44].

Another notable feature in a tissue stress-strain relationship is the change in hysteresis loop shape following several repeated cycles of loading and unloading the tissue. After a certain number of repeated cycles, the shape and the values stabilize [40]. An example of that is seen in Figure 6, in data extracted from the porcine bladder.

As Zanetti et al., 2012 [44] has proven the effect of frequency of preloading and loading history creates significant changes in the mechanical behaviour. With increasing numbers of cycles, the loss of linearity in the hysteresis loop and a more pronounced relaxation are observed [44]. Therefore, it is important to describe the preconditioning cycles in study protocols [21,45]. However, it is important to consider that this behaviour is not native to the tissue either. A normal person urinates six to eight times in 24 h, making it a much lower frequency than the one tested during preconditioning cycles. Too high of a frequency experienced by tissue during laboratory preconditioning cycles might lead to increased stiffness at the initial phases of stretching and decreased stiffness in the final stages of stretching, caused by tissue exudation and micro-failures, respectively. This effect was suggested to be caused by not giving enough time for tissue recovery, leading to skewed effects [44]. Thus, more testing on the optimal preconditioning should be done to accurately describe the native properties.

Tissues can be described by numerous simple properties, such as ultimate stress and strain. However, these quantifications do not reflect the complexity of the tissues and further examination of creep, stress relaxation, hysteresis loops and preconditioning should be employed.

### 4.6. Mechanical Testing

To investigate the biomechanical properties of the bladder and tissue-engineered materials multiple mechanical tests can be employed. The most commonly employed types of mechanical testing are:

#### 4.6.1. Uniaxial Tests

**Figure 7:**
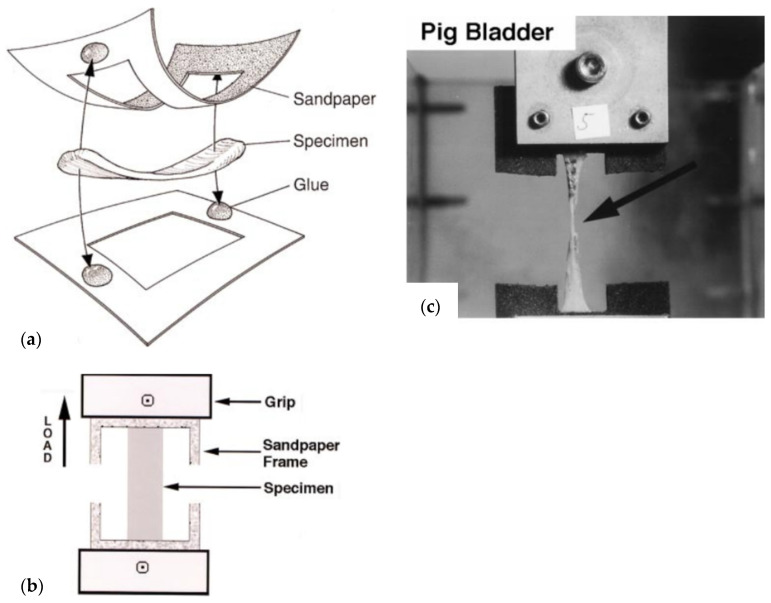
Shows the uniaxial test. Figure obtained with permission from [47]. (**a**) Shows suggested mechanism of sample preparation (**b**) Shows a schematic of uniaxial testing procedure. (**c**) Shows an image of pig bladder during uniaxial testing. Arrow indicates material failure.

The most basic test—the uniaxial test, involves loading (stretching) a strip of tissue or material in one direction. The specimen tested is usually clamped by the edges of the tissue and stretched by the equipment, as shown in Figure 7 (i.e., tensile testing machine). The increasing load is applied until tissue rupture, which is confirmed by the loss of load and tear in the tissue. The load and elongation are recorded throughout the test [46]. That can be used to derive the ultimate tensile strain and ultimate tensile strength, using equations for strain and stress and the values at the point of rupture. If the tissue is cyclically loaded and unloaded below the limits of ultimate stress and strain, the hysteresis loops can be derived.

To investigate the mechanical properties, previously collected data were extracted from uniaxial experiments, shown in Table 1. The data observed are limited and shows great differences between human, porcine and rat bladder. These differences are also confirmed by differences in histological assessment performed by Dahms et al., 1998 [47]. The analysis of human bladders revealed a higher amount of collagen type III and elastin, as well as lower amounts of collagen type I in human-sourced samples compared to both rat and porcine samples. Therefore, to improve the accuracy of the field a consensus on biological models and testing criteria should be developed. Additionally, beneficial would be reaching an agreement to which model organism the developed biomaterials’ characteristics should be matched. Matching the biomaterial to the human biomechanical properties might create a mismatch in the model organism, such as a rat or a pig, which would not indicate the inadequacy of the material to the function in humans.

**Figure 8:**
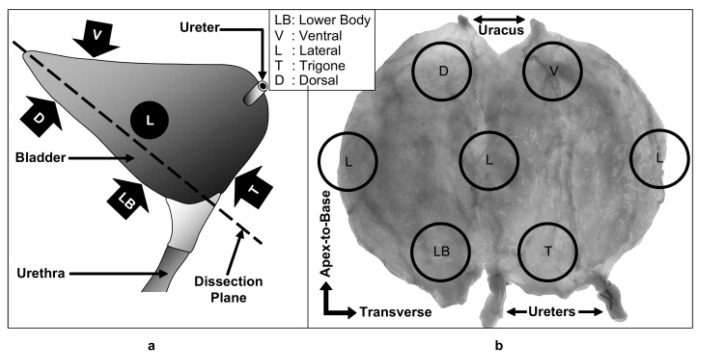
Shows locations of samples obtained. (**a**) Anterior-posterior plane diagram of the bladder indicating the sample localization. (**b**) Cut-opened porcine bladder showing sample localisation. Directions indicated where apex-to-base is also considered as longitudinal and transverse direction also considered as circumferential. Figure obtained with permission from [48].

**Figure 9:**
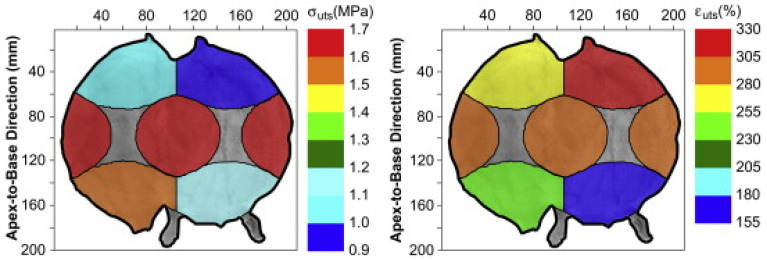
The figure illustrates the biomechanical parameters in different bladder regions. The graphs illustrate the ultimate tensile strength(σ_uts_) and the highest failure strain (ɛ_uts_) mean values obtained from different bladder regions corresponding to Figure 8b. Figure obtained with permission from [48].

Another consideration to obtain accurate and comparable results is the methodology by which the tissue is collected. Different experiments follow different protocols, and some did not indicate the site of tissue collection or the direction in which the uniaxial test was performed. That is particularly important due to the important implications of the site collection on the mechanical properties. That was in-depth investigated by Korossis et al., 2009 [48], who investigated each of the five distinct sites of the bladder separately, as shown in Figure 8 and Figure 9. Each of the parts showed distinct biomechanical qualities depending on the region and direction of the stretch. The augmentation cystoplasty is usually performed in the ventral part of the bladder [52], as seen in Figure 8, therefore this bladder part will be focused on in this section. This region showed the highest strain in the apex-to-base direction, as shown in Figure 9, compared to other parts. That suggests matching the property to enable the stretch is important in this region. The study results are summarised in Figure 9.

The study additionally investigated selected histological features. The ventral region along with dorsal and lateral had shown the highest elastin contents and more compacted muscle bundle regions compared to lower body and trigone regions. Considering the fact that elastin allows for recoiling mechanism in tissues frequently under deformations [53], the conclusion that ventral, dorsal and lateral regions are mostly responsible for distension can be reached. This requires sufficient mechanisms that prevent the permanent deformation of these regions.

These findings overall supported the anisotropy of the bladder tissue, meaning the biomechanical properties depend not only on the sample location but also on the direction in which the force is applied. This property was later confirmed by studies by Chen et al., 2013 [54], Jokandan et al., 2018 [51] and others [44,55]. While Chen et al., 2013 [54] observed a similar trend in the direction of anisotropy, other studies by Parekh et al., 2010 [55] and Zanetti et al., 2012 [44] showed the opposite. These studies are difficult to compare as they showed different methods of testing (uniaxial, biaxial and others), as well as different sample handling methods and differences in the origin of samples themselves (rats and porcine tissues). As an example, the studies showing the opposite trend in anisotropy were performed on rat models. Therefore, while comparing the results, the higher similarity of porcine tissue to the human bladder, based on histological and mechanical test results, should be taken into consideration [47,51].

Additionally, as Jokandan et al., 2018 [51] suggested the discrepancies can be also contributed to different rates of deformation of the tissues. A lower rate of deformation was suspected to lead to low anisotropy, while higher rates were associated with higher anisotropy, where circumferential direction showed higher stiffness compared to longitudinal, directions as seen in Figure 8. Overall, the findings supported that the bladder shows isotropy in strains lower than 200% and above this limit circumferential direction showed higher stiffness [48,51,56]. Taking into consideration that the force exerted on the bladder is its filling with urine, which has a rate of 1–2 mL/min [57], the conclusion that in its natural environment, the bladder shows predominantly isotropic qualities until 200% of strain is exerted on it can be reached.

Overall, limited data on the mechanical qualities of the human bladder exist in the current literature. Additionally, the effect of anisotropy and its biological relevance might make it harder to reach an agreement in the field.

#### 4.6.2. Biaxial and Ball Burst Tests

**Figure 10:**
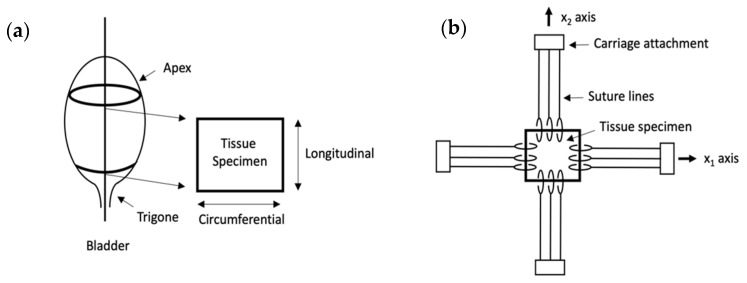
Shows biaxial testing. (**a**) Shows sample collection and preparation. (**b**) Shows machine mechanism.

Uniaxial testing is an easily comparable technique; however, it does not characterize the qualities of anisotropic tissues. Therefore, to understand the full range of bladder biomechanical properties, this tissue should be tested using multiaxial tests. One type of this test is a biaxial test. To perform it the rectangular-cut specimen is hooked by its edges, which are connected by threads to force distributors, which collect the force measurements. Additionally, black marks on the tissue allow to take dimensional measurements, used to calculate the strain of the tissue. The mechanism is shown in Figure 10.

These tests were argued to closely mimic the bladder physiological environment, as naturally three loads are being implemented on the sample. These are two stresses in two axial directions and a transmural pressure load [21,54]. For the bladder tissue, the biaxial tests were mostly done to establish stress relaxation [42,43,58]. These were followed by mathematical efforts to develop better predictive models that would also quantify the contribution of the smooth muscle layer and ECM to the stress relaxation curves [42,43].

**Figure 11:**
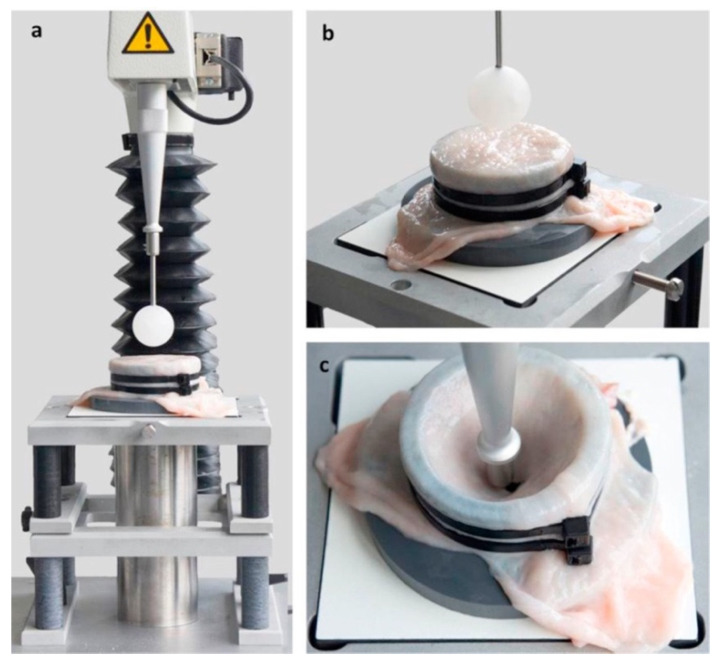
(**a**,**b**) Shows the ball burst test. The tissue is mounted on a cylinder using pull-tight straps. (**c**) Shows the test upon failure of the material with the ball that advanced through the tissue rupturing it. Figure obtained with permission from [51].

Another multidirectional test is a ball burst test. This technique requires a less high-tech set up to achieve similar results and is easier to conduct. The simplicity relies on taking measurements from a rigid ball, which is applying the force on the specimen until it punctures. A sample set up is shown in Figure 11.

Similarly, to the biaxial test, this technique better corresponds with forces naturally exerted on the bladder tissue compared to the uniaxial testing. However, the setup of the machinery makes it harder to analyse the raw ball burst test data. This difficulty in analysis leads to most of the studies focusing on developing mathematical methods useful for the derivation of desired qualities from the raw data [51,59,60]. The studies enabled us to derive multiple qualities, such as stress to failure, failure resultant, ball-burst pressure, maximum elongation [59,60], bladder wall stress and average strain [51].

Only one study to date has performed this analysis on fresh tissue [53] and not ECM [59,60,61,62]. In the study, Jokandan et al., 2018 [51] tested the fresh porcine bladder using multiple techniques, such as uniaxial and ball-burst tests, with results shown in Table 1. These tests and results were suggested as new reference points and the gold standard for measuring the biomaterials qualities for their accurate comparison to bladder tissue. However, not many studies have employed the ball burst tests on bladder wall and biomaterials, thus comparisons so far are hard.

### 4.7. Urodynamic Testing

Previously mentioned tests are mostly carried out on a small sample of the bladder tissue. This is a valuable approach to employ for testing intrinsic bladder properties and biomaterials characterization, however, it does not include the dynamics of the whole organ. To accommodate for the whole organ changes urodynamic tests can be performed. These are a broad range of tests, where the catheter fills the bladder with warm liquid and the intravesical pressure (in the bladder), abdominal pressure and the liquid in the bladder are measured [63]. These measurements can be used to measure the bladder volume, detrusor pressure and compliance [63,64].

Compliance is meant as the ease with which the bladder will extend. It can be determined by the change in volume to the change in detrusor pressure. The normal bladder compliance in women is close to infinity until full capacity is reached. This happens because the increase in volume causes a minimal change in detrusor pressure thanks to detrusor relaxation, as described below [65]. The compliance is restrained by the muscle layer and increases upon its removal from the specimen. However, the role of ECM in bladder tissue should be also considered crucial in providing mechanical qualities. As an example fibrillar collagens present in lamina propria provide strength, structure and compliance, therefore, leading to important mechanical qualities and the ability to regain previous structures [36]. Additionally, the decellularised ECM has similar tensile strength and maximum strain to its cellularised counterparts [47].

The bladder volume and pressure correlation can describe the bladder tone during the filling cycle. This mechanism is essential to the neural control of micturition (voiding). During filling from 0 to 50 mL the rise in pressure is mild, then from 50 to 300 mL, almost no pressure increase is seen because the detrusor relaxation recompensates for volume change. Above 300 mL the pressure increase is steep to the point it signals a need for voiding to the brain. The bladder pressure sensing happens unconsciously until the need for voiding is signalled to the brain at which point voluntary control can inhibit the neurons responsible for stimulating the detrusor muscle to void. This action will be inhibited until we consciously decide that it is socially acceptable to void [66].

The described mechanism strongly depends on pressure sensing by activating stretch receptors in the bladder [66]. If the elasticity of the bladder is impaired due to a mismatch between the bladder wall and material implanted, this mechanism can potentially lead to voiding dysfunctions. Therefore, it is crucial to match the biomaterial to the native qualities of the bladder.

### 4.8. Summary

The mechanical tests described above are currently used to characterise the bladder wall. These measurements function as comparison values to the biomaterials developed. As described previously, multidirectional mechanical tests, such as ball burst testing, more accurately depict the native bladder environment. However, due to the novelty of this technique, not much is known on the human bladder qualities derived from this testing and not many studies use it to characterise their biomaterial. Therefore, in this review alternative measures of uniaxial tests and urodynamic tests are employed to compare how biomaterials address the bladder biomechanical properties.

## 5. Biomaterials for Bladder Reconstruction

**Figure 12:**
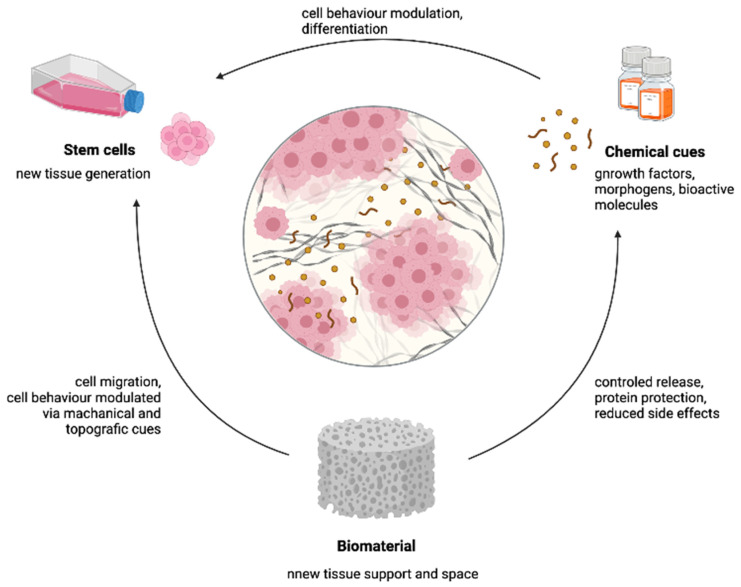
Shows the influence of individual components employed in tissue engineering and their influence on each other. Created using biorender.com (accessed on 14 April 2021).

As described previously, the use of ileal segments for bladder reconstruction surgeries is associated with multiple morbidities [2,7]. For this reason, alternatives to ileal segments were searched for by following the principles of tissue engineering. This approach includes creating devices that will support and foster new tissue growth. Important components of this approach are the cells, the cues and the support for tissue regeneration provided by the scaffold, as represented in Figure 12. The scaffold’s role is to provide mechanical and spatial properties that can guide stem cell differentiation, as well as provide space for the growth of new tissue and maintain organ function during tissue regeneration. The latter is particularly important for load-bearing tissues, such as the bladder [21,67].

The tissue engineering approach provides a necessary improvement for bladder reconstruction surgeries [3,9,21]. However, current research does not live up to the expectations of the community, as none of the materials under clinical trials provided significant benefit for the patients nor have been commercialised [3,9,21,68]. This is caused by many challenges in the vascular supply, cell sources, neural regeneration and mechanical properties of the created biomaterials [9]. This section will focus on evaluating how currently developed biomaterials address bladder biomechanical properties.

To summarise the points mentioned previously, there are multiple reasons to match the mechanical properties of the scaffold to the ones seen in native tissue. Firstly, to ensure normal function upon biomaterial implantation. This is associated with enabling cyclic distension and contraction while maintaining the strength and impermeability of the construct. Additionally, exhibiting and relaying the right stretch signals to the rest of the bladder will enable healthy micturition patterns. Secondly, mechanical stimuli can foster the proper regeneration of the tissue [38].

Currently, most of the research is focused on using material chemistry in addressing mechanical properties. Different material sources for scaffolds have been tested with the aim of providing the desired qualities. These materials can be either natural or synthetic in origin.

Natural materials can be divided between decellularised tissues and materials produced from natural polymers. Decellularised scaffolds usually act as a mechanical support and include native moieties that promote cell attachment and migration [69]. Often used for regeneration of the bladder wall is small intestine submucosa (SIS), which consists of ECM from porcine submucosa and bladder acellular matrix (BAM), which is a 15 decellularised bladder wall. Another option is the creation of materials using polymers of natural origin, such as collagen or silk fibroin. Multiple techniques, such as electrospinning, solvent-casting and extrusion can be later used to fabricate the materials. This approach enables to maintain control over the structure and chemical components of the biomaterials while promoting cell attachment and other biocompatible properties.

Synthetic scaffolds for bladder engineering are mostly made of known organic polymers with degradable properties. Depending on the chemical content the biological and mechanical properties of the scaffold will vary. The bladder engineering studies often employ materials, such as PGA, PLGA and PCL, which show thermoplasticity—a feature enabling usage of previously mentioned fabrication techniques.

Based on the previous data collected on the fresh samples, seen in Table 1, the range of values for native tissue, regardless of the origin, was established to be 0.13–1.2 Mpa. Only twelve out of twenty-nine biomaterial samples showed ultimate stress values within this range, marked by orange highlight in Table 2. Out of them, the majority was created using naturally sourced silk fibroin. This might be caused by silk fibroin having native properties similar to those of the bladder wall. This can be concluded from the cast film sample, which is an even layer of silk fibroin and was determined to have similar mechanical properties to the bladder wall [24].

However, this survey should not conclude that only silk fibroin is capable of creating a suitable material for the bladder wall scaffold. This is not evident in this approach due to a limited number of studies that perform mechanical properties quantification compared to the studies created on the subject. Many other promising materials, such as BAM or SIS have been developed, however, their success was shown by urodynamic testing. This greatly hinders this analysis, as discussed later in the review.

The only study that uses a synthetic material and was able to recreate the biomechanical qualities seen in the bladder wall was conducted by Yao et al., 2013 [77]. The scaffold was designed by creating a nanostructured surface and functionalisation with peptides to promote tissue-forming cell functions. Additionally, the scaffold was made of seven layers with different ratios of PU and PLGA, with an impermeable layer closest to the bladder lumen. Results of the study showed increased in vivo bladder regeneration with fully formed tissue in a minipig model 11 months after implantation. This shows the importance of considerate scaffold design and the possibility for synthetic materials to promote enhanced tissue regeneration.

It can be also observed that the addition of synthetic fabricated materials leads to higher tensile strength. That can be seen by the addition of increasing PEUU content in the electrospun mats was correlated with the increased tensile strength of the materials [72]. Similarly, the addition of synthetic materials, in the forms of nanofiber mat or knitted mesh, to a naturally sourced collagen significantly increased the tensile strength of the constructs [73,74]. This hybrid scaffold fabrication was also seen beneficial for BAM where deposition of electrospun mats on top of the BAM enabled to maintain normal filling volume compared to extensive distension and pathological increase in bladder volume in BAM scaffolds without nanofibers [80], as measured by urodynamic studies. Therefore, the combination of both synthetic and natural materials can enable adjusting the biomechanical and biological properties to the ones required for bladder bioengineering.

### 5.1. Pre-Seeding Cells into Scaffolds

Another key consideration is the addition of cells to the construct. In Table 2 it can be seen that the addition of cells increased the ultimate tensile strength of the constructs [50,76]. That was apparent based on the difference in pre and post-operational values of the scaffolds, 0.05 ± 0.03 MPa and 0.14 ± 0.06 MPa in comparison to 0.26 ± 0.02 MPa and 0.25 ± 0.03 MPa respectively [50]. Additionally, the increasing time of cell culture on the scaffold also increased the tensile strength of the material [76]. These results are consistent with the finding by [81], which found SIS seeded material was stiffer after 20 days of cell culture, as measured using biaxial strain measures.

This behaviour can be overall associated with cell ECM deposition. This can be concluded from greater amounts of collagen found in the study by Sivaraman et al., 2015 [76]. This amount was increasing with longer periods of cellular culture. Similarly, the study by Tu et al., 2013 showed the tissues established new urothelium, lamina propria with extensive ECM layer and smooth muscle bundles after implantation. This ECM altering behaviour was also seen by Lu et al., 2005 [81], which suggested that in the first 10 days of cultures the cells constituted to fast ECM remodelling of SIS biomaterial. This shows that cells can potentially increase the strength of the construct. This aspect should be included in the biomaterial design for bladder wall regeneration.

Designing softer materials to compensate for the cell ECM deposition is a controversial idea compared to other approaches reported [21]. This study considers the stiffening effect of the cells to be smaller than the predicted softening upon implantation due to biomaterial degradation. However, the opposite trend is seen in urodynamic studies of SIS biomaterials. Even though the native properties of material show lower strength compared to the native bladder, as seen in Table 2, many scaffolds experience a decrease in compliance [20,82,83,84,85]. This can be explained by extensive scaffold fibrosis [86]. Therefore, more investigations should address the question of whether we should include the stiffening behaviour of the cells and fibrosis or the softening mechanism of degradation when designing the biomechanical properties of the scaffold. Overall, a prediction can be made that the ideal biomechanics would depend on the scaffold, the cells, the age of the patient and others [21]. Therefore, the approach to the biomaterial mechanical qualities might differ depending on the intervention being made.

To date, the influence of scaffold remodelling has not been explored in detail. It is, therefore, necessary to further explore its role in changing the mechanical properties of the biomaterials and the mechanisms associated with this behaviour. These findings could change the values of the mechanical properties the field of biomaterials for bladder engineering aims to achieve.

### 5.2. Quantification of Biomechanical Properties

The presented biomaterials should not be judged only on their ability to match the mechanical properties of the native bladder. That is because the sole comparison of quantified properties doesn’t give a complete understanding of their viscoelastic properties, as explained previously. That was the case for plastic compressed collagen, developed by Ajalloueian et al., 2013 and Ajalloueian et al., 2014 [73,74], which matches the ultimate tensile strength value to native tissue, however, the construct stability is poor. The construct was not capable to maintain its structure and shape, when hold, and experienced intense shrinking upon cell seeding.

**Figure 13:**
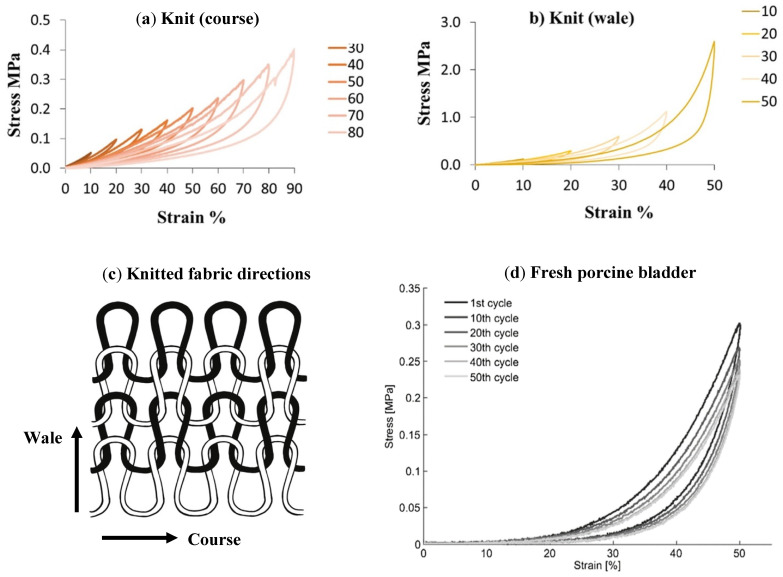
Shows the loading-unloading curves for knit material and fresh porcine bladder. (**a**) Shows the curves obtained from knit tested in the course direction. (**b**) Shows the curves obtained from knit tested in the wale direction. (**c**) Diagram shows the knit directions. (**d**) Shows the curves obtained from fresh porcine bladder. (**a**,**b**) obtained with permission from [24], (**d**) obtained with permission from [44].

The opposite of this behaviour is seen in knitted silk fibroin material developed by Khademolqorani et al., 2021 [24]. The cyclic tensile tests showed a hysteresis loop resembling the ones of the native bladder, as seen in Figure 13, however, the construct’s ultimate tensile strength was around ten times higher than the one shown in native tissue, 9.4 ± 1.3 MPa compared to 0.13–1.2 MPa respectively. This shows the need for a more profound analysis to be employed in order to determine the suitability of constructs for bladder bioengineering and their viscoelastic properties.

Unfortunately, many studies currently performed do not employ the tests needed to establish the viscoelastic properties. These as mentioned above would require extracting the loading and unloading curves from cyclic tensile tests. The lack of employing these tests makes it harder to perform an in-depth analysis to establish the suitability of these materials for the replacement of viscoelastic tissues.

This lack of quantification is seen not only in the cyclic uniaxial tests but also in the mechanical tensile testing overall. As seen in analysis by Ajalloueian et al., 2018 [21], most studies employ urodynamic tests upon biomaterial implantation into an animal model. This analysis enables to perform whole organ study but might not give enough insight into which qualities might contribute to the success or the failure of the scaffold. Whole organ tests are usually employed by studies focusing on naturally sourced biomaterials, such as BAM or SIS [21]. Analysis of the mechanical properties of the biomaterial and its contribution to regaining the appropriate bladder volume after augmentation procedure is hindered by not using both types of mechanical testing in singular studies. Employing these tests would allow for understanding which mechanical qualities might be important to regain normal bladder capacity.

### 5.3. Urodynamic Studies on Biomaterials

As mentioned previously, many studies do not employ mechanical tests, therefore, the above analysis might present a limited view on the biomaterials used in the field. Many studies that employ natural materials perform urodynamic studies, thus in this section, analysis of the promising biomaterial approaches that lack quantification in mechanical properties will be performed.

One of these materials is the small intestine submucosa (SIS). Unfortunately, many studies show shrinking and contraction of the material upon implantation into the animal models [20,82,83,84,85,87,88,89,90,91,92,93]. This approach has also failed trials in humans [20,82], by showing an unsatisfactory increase in bladder capacity and compliance, as well as poor tissue regeneration, related to fibrosis, as mentioned before. Further work on changing the pro-fibrotic immune response would be beneficial in improving this material’s suitability for reconstruction surgeries.

Another material tested using urodynamic studies is the bladder acellular matrix (BAM). Mechanical tests performed on BAM showed similarities in biomechanical properties regardless of the BAM scaffold origin [47], as seen in Table 2. Additionally, its stability [94] and maintaining of native tissue architecture would suggest the suitability of BAM for reconstruction surgeries [95]. This prediction was confirmed by studies showing the recovery of normal bladder volumes upon implantation of the BAM scaffold [75,96,97]. Additionally, the role of cells was found to be significant in preventing shrinkage and supporting smooth muscle growth [94,95]. Further developments in addition to growth factors and appropriate cell sources are expected to make BAM a suitable alternative for ileal segments [98].

However, the comparison of the urodynamic studies is difficult as big heterogeneity in the study designs is present [99]. A systematic review performed by [99] determined that it is possible to regain normal bladder volume after the augmentation procedure using a biomaterial. However, this outcome might depend on the animal model being used, as dogs and pigs were depicted as more representative. Unfortunately, the effect of different types of biomaterials was not analysed. In this review, it was proven that the type of the biomaterial should have a profound impact on the mechanical properties and therefore, bladder volume. Thus, an analysis showing the significance of the type of biomaterial on urodynamic testing should be performed.

### 5.4. The Importance of Scaffold Structure on Successful Outcomes

As mentioned previously most of the research is focused on addressing the biomechanical properties using different materials for scaffold creation. Unfortunately, the expectations of the field were not matched by single-material constructs, therefore, many hybrid scaffolds are currently developed [24]. However, the mechanical properties can also be addressed by multiple structural approaches, as discussed in this section.

A study by Khademolqorani et al., 2021 [24] showed that structure is as important as biochemical considerations. It was demonstrated that the structure of the biomaterial, which was a result of different fabrication methods, had significant implications on its mechanical properties, as seen in Table 2. Silk fibroin was used to produce cast film, electrospun fibres and knitted material. The tensile strength was found to be the lowest in cast film and the highest in knit scaffold. Interestingly, anisotropy and different loading-unloading curves of the knit material were found by performing the tensile tests in wale and course directions of the knitted materials, as shown in Figure 13. Additionally, the knit scaffold showed superior qualities in cell interaction and compliance. Similarly, in a study by Tu et al., 2013 [50] the addition of extra film casting on top of a porous material also increased the stiffness of the material, as seen in Table 2. This shows that structural considerations should be explored in greater detail to create an alternative pathway in achieving the desired biomechanical qualities.

Another function of the biomaterial structure should be the preservation of the barrier function usually performed by umbrella cells. As mentioned before, the lack of uroplakin leads to various renal dysfunctions. This situation shows similarity with the scaffold porosity that enables the urine to leak into the structure. Interestingly, scaffold porosity was associated with development of fibrotic tissue in muscle fibre layer and consequently scaffold contraction [23]. Therefore, adjusted permeability that would protect against toxicity, but enable cell infiltration and migration should be pursued in biomaterials. An example of this scaffold structure could be the addition of impermeable film on a porous scaffold, as discussed previously [50,77].

Structural considerations can also impact other properties, such as degradability, stem cell differentiation and material anisotropy [67]. All of these are important challenges in biomaterial design for bladder wall [3,9,21]. Therefore, more research should be performed in order to optimise the materials used with the consideration of structure to improve the biomaterials outcome.

## 6. Conclusions and Summary

Many conditions can affect bladder function which are currently treated using bladder reconstruction surgery [2,3]. These interventions aim to restore urine storage and voiding function often by implanting ileal segments into the bladder wall. These surgeries are associated with co-morbidity that significantly lowers the quality of life of the affected person [2,6].

Tissue engineering offers a promising approach to create constructs that can be designed to suit the properties of the replaced organ [9]. Multiple materials have been proposed for use in tissue engineered bladder constructs; however, none of the clinical trials to date have been successful [23]. This is largely due to underestimation of the bladder wall complexity that provides a protective barrier against toxic urine and contributes to its mechanical properties associated with constant distension and shrinkage [23]. Therefore, the field needs to further investigate the properties that enable the bladder to fulfil these functions. This review focused on the mechanical qualities required to achieve a likely successful tissue engineered bladder construct.

Biomechanical properties are a significant aspect of the scaffold biomaterials as they enable the function to be maintained during regeneration and can support the regeneration process through mechanical cues [67]. The biomechanical properties of the bladder can be described by simple measurements (stress, strain) as well as more complex measurements, associated with tissue viscoelasticity. Overall limited data of mechanical tests makes it harder for the field to reach a consensus on the native properties of the bladder. Reaching a consensus on this matter can be further restrained by the anisotropy of bladder tissue, which is associated with various controversies regarding the direction of anisotropy and its biological relevance.

Most of the current biomaterials that are tested using tensile testing fail to recapitulate the bladder wall mechanical properties. This is linked to the lack of reference values agreed upon by the field. However, this might not depict the situation of the field truthfully because many biomaterial studies simply lack mechanical quantification to perform a profound analysis.

More studies are needed to understand the impact of tissue growth and remodelling on the mechanical properties of the scaffold and the mechanisms that drive these changes. Additionally, the field would benefit from considering the scaffold structure as an inherent and important part of the tissue engineered construct that could facilitate protection of the tissues. This could also enable refining of the mechanical properties without compromising the chemical composition of the material. Creating study protocols that would require high-quality data from both mechanical and urodynamic tests would enable us to reach a conclusion on which mechanical qualities contribute the most to restoring normal bladder capacity. This would provide substantial answers to address the challenges of the field.

Apart from challenges in the biomechanical properties of bladder engineered scaffolds, several crucial challenges associated with different scientific disciplines need to be addressed to facilitate the commercialisation of bladder engineered constructs. These include vascularisation of the construct [100], neuronal network integration [101], identifying an optimal source of stem cells [102,103], predicting long-term mechanical properties of the construct [21] and controlling the immune response to the implanted tissue engineered construct [9]. These multiple problems can only be addressed by collaboration between multidisciplinary teams composed of experts within these fields.

The field should aim to reach a consensus on the key biomechanical properties and the optimal range of values. This would provide target values for which new scaffolds under development should exhibit. Following this, the field could establish a more coherent database on the mechanical properties and the consequent capacity of the bladder scaffolds. Furthermore, this approach would provide clarification on the unmet needs in the field as well as highlighting the importance of biomechanical properties in restoring bladder function.

## Figures and Tables

**Table 1:** Represents data extracted from the previously performed studies. All the results were obtained from uniaxial testing, besides the values marked in green. This data was obtained from a ball burst test and illustrates the differences between the tests as well as acts as a reference point for other data collected.

Author	Samples		Ultimate Tensile Strain (%)	Ultimate Tensile Strength (MPa)
Dahms et al., 1998 [47]	Different bladders	Human	69 ± 17	0.27 ± 0.14
		Pig	166 ± 31	0.32 ± 0.1
		Rat	203 ± 44	0.72 ± 0.21
Korossis et al., 2009 [48]	Porcine Bladder	Ventral	350 ± 50	0.9 ± 0.2
		Dorsal	290 ± 30	1.0 ± 0.2
		Lateral	290 ± 30	1.1 ± 0.1
Martins et al., 2011 [49]	Human female bladder	Bladder dome (no direction indicated)	-	0.9 ± 0.1
Tu et al., 2013 [50]	Fresh Porcine Bladder	No site indicated	700 ± 10	0.21 ± 0.04
Jokandan et al., 2018 [51]	Fresh Porcine Bladder	Circumferential	435 ± 69	0.28 ± 0.03
		Longitudinal	358 ± 21	0.34 ± 0.014
		Ball burst	389.9 ± 58.9	1.45 ± 0.39

**Table 2:** Represents data extracted from the previously performed mechanical tests on biomaterials. All the results were obtained from uniaxial testing. The values matching the native properties of the bladder wall are highlighted in orange.

Author	Scaffold Type	Regeneration Site	Cells Seeded	Results	Sample Type	Ultimate Tensile Stress (Mpa)	Ultimate Tensile Strain (%)
Qiu et al., 2019 [70]	SIS	none	none	The actual mechanical properties of sis can differ depending on animal age [71].	SIS	0.05 ± 0.0017	470.3 ± 1.93
Stankus et al., 2008 [72]	urinary bladder matrix and PEUU electrospun mats	subcutaneous injection	vascular smooth muscle cells	With higher PEUU content higher tensile strength and strain were observed, however more PEUU was associated with higher inflammation.	PEUU content 25%	2 ± 0.1	40 ± 0.6
					PEUU content 50%	4.9 ± 1.6	83 ± 31
					PEUU content 75%	11.8 ± 0.7	143 ± 10
					PEUU content 100%	12.9 ± 1.7	220 ± 77
Ajalloueian et al., 2013 [73]	hybrid of plastic-compressed collagen with PLGA electrospun fibres	Petri dish	the minced pig bladder mucosa	Increase in tensile strength for the hybrid scaffold, the cells from mince infiltrated the construct and after 4 weeks formed urothelium typical to urothelial histology.	Plastic-compressed collagen	0.6 ± 0.12	5
					Hybrid of plastic-compressed collagen and PLGA nanofibers	3.57 ± 1.1	81
Ajalloueian et al., 2014 [74]	hybrid of plastic-compressed collagen with PCL-knitted material	petri dish	the minced pig bladder mucosa	Both scaffolds support the growth of the urothelium. The hybrid showed higher tensile strength and remained stable while the collagen significantly contracted.	Plastic-compressed collagen	0.6 ± 0.12	5
					Hybrid of plastic-compressed collagen and PCL knitted mesh	17.9 ± 2.6	67
Zhao et al., 2015 [75]	hybrid of porous silk fibroin and BAM graft	rat bladder	none	No significant loss of tissue response or systemic toxicity. The material supports regeneration. The procedure increases bladder capacity compared to the control group.	BAM-silk fibroin hybrid scaffold	0.39 ± 0.09	88.17 ± 18.16
Sivaraman et al., 2015 [76]	composite hydrogel Tetronic (BASF) 1107-acrylate (T1107A) blend with collagen and hyaluronic acid	petri dish	bladder smooth muscle cells	The constructs were showing higher stiffness the more time the cells were cultured on the scaffold. The study hypothesized that prolonging the culture might lead to matching properties to the bladder.	acellular	0.0041 ± 0.0012	121 ± 123
					cellular 7 days	0.0052 ± 0.0006	123 ± 4
					cellular 14 days	0.0116 ± 0.0022	139 ± 12
Yao et al., 2013 [77]	PLGA and polyurethane nanoscaffolds composite	minipig model	none	Composite scaffold supported regeneration in a minipig model compared to ileal segments. The engineered scaffold layer was easily separable after tissue regeneration.	PLGA and polyurethane nanoscaffolds composite	0.71 ± 0.15	NA
Tu et al., 2013 [50]	silk fibroin scaffold created using solvent-casting or solvent-casting and silk film casting	Yorkshire swine	none	Animals showed high rates of survival. An increase in bladder capacity and compliance was seen. The scaffold also supported the tissue regeneration with innervation and vascularisation.	solvent-casting pre-operatively	0.05 ± 0.03	30 ± 10
					solvent-casting and silk film casting pre-operatively	0.14 ± 0.06	45 ± 12.5
					solvent-casting post-operatively	0.26 ± 0.02	550 ± 10
					solvent-casting and silk film casting post-operatively	0.25 ± 0.03	300 ± 50
Khademolqorani et al., 2021 [24]	weft knit, cast film and electrospun scaffolds from silk fibroin material	petri dish	mouse fibroblasts	The knit scaffold showed superiority when considering properties important for bladder regeneration, such as lowest stiffness and highest strength and high cell infiltration.	weft knit course direction	7.8 ± 0.9	377.7 ± 15.4
					weft knit wale direction	9.4 ± 1.3	138.7 ± 14.1
					cast film	0.2 ± 0.1	77.7 ± 9.1
					electrospun fibres	1.0 ± 0.2	19.8 ± 3.4
Del Daudio et al., 2013 [78]	electrospun PCL and PHBV blend 50:50	rat model	none	Scaffold showed some regenerative capabilities with urothelium coverage and muscle cell infiltration.	50:50 PCL and PHBV blend electrospun mats	1.4 ± 0.3	270 ± 80
Sivaraman et al., 2013 [79]	PCUU, PGS-PCL and PEUU electrospun scaffolds	rat model	none	The scaffolds were tested using cytocompatibility studies and based on the results the PCUU scaffold was selected for bladder augmentation. Bladder augmentation increased animal survival but was associated with stone formation.	PGS-PCL	0.072 ± 0.005 (N/mm)	215 ± 28
					PEUU	1.75 ± 0.68 (N/mm)	247 ± 52
					PCUU	0.43 ± 0.029 (N/mm)	243 ± 26
Dahms et al., 1998 [47]	rat, pig and human BAM	none	none	The scaffolds show different levels of collagen that comprise the matrix. The differences between the mechanical properties were not found to be statistically significantly different.	rat BAM	0.68 ± 0.21	0.73 ± 0.23
					pig BAM	0.29 ± 0.05	1.86 ± 0.51
					human BAM	0.13 ± 0.05	0.91 ± 0.08

## Data Availability

Not applicable.
